# Alabama Medical University

**Published:** 1845-12

**Authors:** 


### ALABAMA MEDICAL UNIVERSITY.

A medical *university!* That is something new. A university has been considered an institution of several departments—an assemblage of colleges, in which, besides mathematics, the languages, et cetera, medicine, law, and theology are taught. But that is not the notion, it seems, among the law-makers of Alabama. They have chartered a *medical* university, and its Faculty are steam doctors—"purely of the Thomsonian order," as the faculty themselves avow. These *teachers (doctors)* proclaim to the world, "that an epoch in the history of Alabama will *bare* on the first Monday in November, 1845," when the lectures in their *university* commenced. It is an epoch, sure enough. Most signally does it illustrate the boasted "march of mind" in our age. It is a burlesque upon medical schools. One might suppose that the legislature of Alabama was seeking to bring the medical profession into contempt, when they granted such a charter. But it is not so. It was only giving the "largest liberty." Wetumpka is the honored seat of this "medical university," and we shall not be surprised to hear that these doctors of the "Thomsonian order," have had pupils. The legislators who voted for the charter ought to send their sons there. Y.

